# Bibliographical Notices

**Published:** 1846-08

**Authors:** 


ARTICLE X.

*Clinical Lectures on Surgery.* Delivered at St. George's Hospital, by SIR BENJAMIN BRODIE, Bart., V. P. R. S., Sergeant Surgeon to the Queen, &c. &c. &c. Philadelphia: Lea & Blanchard. 1846. pp. 352. (From the publishers.)

Brodie's lectures have been in the course of publication, during the past year, in the "Medical News," and have thus been extensively circulated, and are now issued in a complete state. We have already, from time to time, laid before our readers extracts from them, and may have occasion to do so hereafter. The author ranks, beyond all dispute, among the first writers at present living, and this work is full of practical instruction. No surgeon should be without it. The following observations will not be without interest to our readers:

"When you suspect that pressure on any part is so great as to be likely to occasion mortification, you can do nothing but remove the pressure. When a bandage is placed in a case of fracture, you must remove it as soon as you suspect that the swelling of the parts has made it very tight, lest mortification should follow. When a patient has been so long confined to his bed, that you expect mortification will take place, you must endeavour to guard against it. It is more easy to prevent it than to stop it when it has once begun. How, then, is this to be accomplished? If a patient lies on his back, the skin sloughs over the os sacrum; if on one side then it sloughs over the great trochanter. Endeavour, when you can manage it, to make a patient vary his posture. If he can be shifted, let him lie at one time on his back; at another on his side: nay, let him turn round, and lie occasionally on his face. If you have what they call a prone couch, properly constructed for the purpose, he may, in many instances, use it to great advantage. In one of the worst cases of this kind, when mortification had begun, I used to turn the patient on his face many hours in the day, and with perfect success. But sometimes the patient cannot be shifted. There may be fracture of the thigh, and he must lie always on his back. You must then endeavour to take off the pressure by other means—by an air cushion with a hole in the centre, the tender part over the os sacrum being in the hole of the cushion. But in all cases where you use an expedient of this kind, you should first apply a piece of common soap plaster, spread on calico, over the part, to support it. If you merely place the hole of the cushion under the os sacrum, the skin will bulge into the hole, and the patient will lie as bad as if there were no hole at all, or even worse. The same rule applies to all cases where you use contrivances to take off pressure, as in those of corns and bunions. In cases where you can have resource to it, the water-bed is very useful in preventing mortification from pressure. Dr. Arnott's hydrostatic or water-bed diffuses the pressure everywhere. When you lie on a mattress, the pressure is thrown on all the prominent parts of the body, and little elsewhere; but in using the water-bed the water rises to fill up the hollow places, and the pressure is not greater on the sacrum than on other parts. No doubt this bed is the best method which has yet been contrived for preventing mortification from pressure—the only objection to it is, that it is not applicable to all cases. In cases of compound fracture of the thigh or leg, for example, it would not give sufficient steadiness to the injured limb.

"But another plan may be adopted to prevent mortification from pressure—that is, to prevent the inflammation which precedes it. The thicker the cuticle the more it will protect the parts beneath it. You may, if you attend to it in time, add to the thickness of the cuticle by stimulating the surface of the skin. Nurses know this very well, for when patients are bed-ridden, they wash the parts subjected to pressure with brandy. What is better, is a lotion composed of two grains of oxymuriate of mercury to an ounce of proof spirits. When you think that a patient is likely to be confined so long in bed that there may be mortification from pressure, wash the parts two or three times a day with this lotion. I have found it useful in other cases where a patient suffers from pressure. A man has a rupture which requires to be supported by a very powerful truss. It galls and frets the skin, and may at last cause inflammation and sloughing; but under the use of the lotion, a thicker cuticle is generated and this mischief is avoided."—p. 63.

"It has been a sort of *dictum* of the schools of surgery, that you should not amputate while mortification is going on; and certainly, when there is mortification from ossified arteries (as I shall hereafter explain,) or when there is mortification from inflammation, you ought to wait for the mortification being stopped, and for the formation of a distinct line of separation, before you proceed to an operation. But it must have been palpable to every body who took the pains to consider the subject, that this rule would not apply to all cases of mortification. For example, a man has a strangulated hernia; when you open the sac you find the omentum strangulated, a part of it dead, and the mortification still extending. You would not hesitate in a case like this to cut off the dead and dying omentum. If piles were undergoing the process of mortification from being strangulated by the sphincter muscle, you would not hesitate to cut them off. You may conceive many other cases, in which the cause of mortification is local, and to which the general rule which I have just mentioned does not apply. Baron Larrey has the credit of having pointed out more distinctly than had been done before, that where there is mortification from local injury, you may venture to amputate, though the mortification is still spreading. But I apprehend that the operation must be had recourse to at once, and that the case admits of no delay. If, in consequence of local injury to a limb, mortification has begun, but has not yet produced any severe shock on the system, there you may amputate. But where the mortification has been going on for some days, so that the system has begun to be influenced by it, the pulse getting weak, perhaps intermitting, and with great prostration of strength, in such a case you must not venture to amputate. Under such circumstances it is probable that the system is not in a state to bear the additional shock of the operation. However, I believe that cases enough may be adduced to prove that Baron Larrey's rule of not waiting to amputate till the mortification has stopped, is applicable in a great number of instances where the disease arises from local injury. It is good in theory, and there is now sufficient experience to enable us to say that it is good in practice also." —p. 66.

---

ARTICLE XI.

*The Practice of Surgery.* By JAMES MILLER, F. R. S. E., Professor of Surgery in the University of Edinburgh, etc., etc. Philadelphia: Lea & Blanchard. 1846. pp. 496. (From the Publishers.)

This work is a sequel to the Principles of Surgery, of which we had occasion, some months since, to express a favorable opinion in this Journal.

Taken together they form a very condensed and complete system of Surgery not surpassed as a text book by any work with which we are acquainted, and well adapted for a book of reference for practitioners.

We subjoin the following which will exhibit the practice of Professor Miller in a case where difference of opinion exists: that of fracture of the neck of the femur *within the capsule.*

"*Union* of this fracture is quite possible, but yet improbable. The following are the more important obstacles to such an occurrence. 1. There is an obvious difficulty in maintaining accurate apposition; restraining splints cannot be applied to the part itself, and it is difficult to maintain a uniform ascendency over the retracting muscles. If the periosteal investment remain partially entire, however, there may be little displacement, and proportionally slight shortening; and, in such a case, a better issue may be looked for. 2. There must be a want of provisional callus; there being no structure from which it may be produced, and in which it may be formed and sustained; the synovial capsule is obviously barren in this respect. The fractured ends may be said to be steeped in an increased secretion of synovia. 3. Also the definitive callus, which, if uninterrupted, might alone achieve consolidation—as happens in other fractures, when from any cause the provisional formation has been aborted—is ever liable to accident, by even slight movement of the parts. 4. The upper fragment, or head of the bone, nourished only through the round ligament, must be of weak power, and ill able to execute the exalted nutritive action necessary for reparation. 5. The age of the patient, and the atrophied condition of the bone itself, are obviously unfavorable to reunion.

"With such adverse complications, it is no wonder that examples of union in this fracture are most rare. And yet circumstances may occur, in which that result may be attempted and expected, with every reasonable prospect of success. When, for example, the patient is comparatively young, when the shortening is slight, indicating but partial division of the periosteal investment, when the patient joins heartily with the surgeon in the use of means calculated to maintain apposition, and to prevent all movement of the fragments; and when neither become weary of the prolonged period of vigilance required,—for, be it remembered, provisional callus is wanting, and the definitive must do all. The ordinary result is the formation of a false joint; the parts becoming accommodated to each other by absorption, connected by new fibrous texture, and farther restrained by a thickened state of the capsular ligament; the limb remaining deformed, and comparatively powerless, yet permitting of tolerable comfort and usefulness, with the aid of a stick or crutch. In the extremely old, fatal sinking is very probable, under the shock of the injury, and the irritation of pain and confinement.

"In the last named class of patients, the use of means for effecting retention of the fragments is not expedient. Success cannot result; the annoyance will but aggravate the general disorder; and, not improbably, sloughs will form at the points where the splint exerts its pressure. It is sufficient to arrange the limb comfortably on pillows; and by very gentle swathing or deligation, to restrain motion. In the more hopeful cases, the long splint is to be applied as in treatment of the following injury.

---

### ARTICLE XII.

*A Clinical Introduction to the Practice of Auscultation and other modes of Physical Diagnosis, intended to simplify the Study of the diseases of the Lungs and Heart.* By H. M. Hughes, M. D., Fellow of the Royal College of Physicians, &c. Philadelphia: Lea & Blanchard. 1846. pp. 270. 12 mo. (From the Publishers.)

The study and practice of auscultation and percussion is at the present day absolutely essential to every physician who would practice his profession to his own satisfaction, and afford to his patients all the relief which medical science can give. Unfortunately, but a small number of graduates in our schools are sufficiently acquainted with these means of diagnosis, to render them practically useful, and hence we find them either going through with these methods of exploration, where required by public opinion where they practice, without deriving from them any useful results, or neglecting them and denying their value altogether.

The reason of this state of things is obvious. It is that there are no means of becoming practically acquainted with them except by the aid of a good teacher, in a hospital. Hence, although there are at present numerous good works on the subject they are not of the value desired except to students in attendance upon hospital practice.

To such and to all desirous of possessing a simple description of the physical signs of disease, we can recommend this work. To those who desire fuller instruction in reference to the pathology of thoracic diseases, the great work of Lænnec, with notes by Andral, and that of Piorry on percussion, are to be preferred. The object of the author, which we think he has succeeded in obtaining, is thus set forth in the preface.

"That object is *not* to attempt to teach the practice of auscultation and percussion;—that, I feel assured, can be attained only at the bed-side of the patient—but it is to point out to the student the way in which he may learn it by himself. It is *not* to treat of the diagnosis of thoracic diseases; upon that I profess not here to enter; but it is to point out the physical signs of those diseases, and, as far as I am able, simply and intelligibly to explain the causes of those signs; it is to instruct the beginner in the mode, by which he is to obtain a knowledge of them, as well as to direct him how to interpret them."

